# Long-Term Benefits of Tailored Exercise in Severe Sarcoidosis: A Case Report

**DOI:** 10.3390/ijerph17249512

**Published:** 2020-12-18

**Authors:** Alba M. Herrera-Olivares, Juan M. García-Manso, Irene Rodríguez-Gómez, Ignacio Ara, Alejandro Lucia, Alfredo Santalla

**Affiliations:** 1 Faculty of Sport Sciences, Universidad Europea de Madrid, 18670 Madrid, Spain; amheroli@gmail.com; 2 Hospital San Juan de Dios, Santa Cruz de Tenerife, 38009 Canary Islands, Spain; jgarciamanso@gmail.com; 3 GENUD Toledo Research Group, Universidad de Castilla-La Mancha, 45004 Toledo, Spain; Irene.Rodriguez@uclm.es (I.R.-G.); Ignacio.ara@uclm.es (I.A.); 4 CIBER of Frailty and Healthy Aging (CIBERFES), 28029 Madrid, Spain; 5 Instituto de Investigación Hospital 12 de Octubre (imas12), 28041 Madrid, Spain; asanher@upo.es; 6 Faculty of Sport Sciences, Universidad Pablo de Olavide, 41013 Seville, Spain

**Keywords:** cardiopulmonary exercise test, pulmonary fibrosis, high-intensity interval training, inspiratory muscle training

## Abstract

Background: We studied the effects of a supervised, structured exercise program in a severe sarcoidosis patient. Methods: After being clinically stable for two years, a 52-year-old woman (stage IV, American Thoracic Society) who originally had irreversible lung fibrosis, pulmonary arterial hypertension (PAH), mild mitral insufficiency, and atrial dilatation, and was candidate for lung transplant, performed a combined high-intensity interval, high load resistance, and inspiratory muscle training for 4.5 years, and was tested (cardiopulmonary exercise testing and dual X-ray absorptiometry) every six months. Results: Cardiorespiratory fitness (CRF) and maximal pulmonary ventilation increased by 44% and 60%, respectively. Ventilatory efficiency also improved (decrease in the ventilatory equivalent for oxygen by 32% and 14% at the ventilatory threshold and respiratory compensation point, respectively). She improved New York Heart Association (NYHA) class (from III to II), and cardiac alterations as well as PAH reversed so that she was not in need of lung transplantation anymore. Likewise, she suffered no more episodes of hemoptysis. Bone health was overall maintained despite the post-menopausal status and the corticoid treatment. Conclusions: A long-term combined exercise intervention safely contributed—at least partly—to improve CRF and NYHA class in a patient with severe sarcoidosis, suggesting a potential coadjuvant effect to attenuate clinical manifestations.

## 1. Introduction

Sarcoidosis is an autoimmune disease of unknown etiology (incidence of 2.17–519/100,000 people/year [1]), characterized by the presence of noncaseating granulomas in the affected organs [2]. The condition is more common in women than men, with a peak age of onset of 30–55 years. There is high individual variability in the clinical manifestations, and many patients are asymptomatic or will have remission of symptoms within two years [3]. Sarcoidosis can affect several organs, but it preferentially affects lymph nodes and the lung where it can cause different levels of damage ranging from asymptomatic involvement to severe manifestations, including pulmonary fibrosis [4]. Pulmonary vessels might also be affected, which increase the risk of a life-threatening complication—severe pulmonary arterial hypertension (PAH) [5].

Fatigue is the most commonly reported symptom of sarcoidosis [3], which together with dyspnea, persistent cough, and physical limitations, can impair the quality of life (QoL) of symptomatic patients and lead to anxiety, depression, and social isolation [6]. Patients commonly have low levels of physical activity (PA) [7,8] and functional capacity [7,9] and, in fact, international experts recommend the implementation of exercise for treating symptomatic patients [10]. However, there is still a lack of evidence as to which training program is best (type of exercise, intensity, frequency, or duration) for this patient population [10]. In general, physical exercise interventions in patients with sarcoidosis have combined aerobic and strength exercises (as well as breathing exercises) for a maximum duration of 13 weeks [11,12,13,14,15]. The typical aerobic component consists of moderate-intensity continuous training (MICT, e.g., 20–30 min of walking/cycling at ≤70% of maximal aerobic capacity). In this regard, a type of exercise training that is gaining popularity in Western societies is high-intensity interval training (HIIT), because it is thought to stimulate aerobic fitness and muscle molecular adaptations that are comparable—if not superior—to those elicited by MICT despite a lower time commitment [16]. However, no study has yet assessed the long-term effects (or “adaptations”) induced by HIIT in symptomatic patients with sarcoidosis, in part because the potential exacerbation of the feeling of fatigue with intense exertion is a concern in this population [12]. Preliminary evidence has, however, indicated that a single HIIT session does not affect fatigue differently from a single MICT session in patients with sarcoidosis, supporting the need for further research on the long-term effects of HIIT for these patients [17].

Approximately 10% of patients with sarcoidosis develop end-stage fibrotic lung disease with high indication for lung transplantation [18]. Prior to this, patients may experience long-term and progressive muscle weakness [19], also affecting the inspiratory musculature [20]. Accordingly, it would be of clinical value to provide preliminary (or “proof-of-concept”) evidence on the potential benefits of programmed exercise performed over the years in those patients with a worse prognosis. Here we present the case of a patient with systemic sarcoidosis and associated severe lung affectation who underwent a supervised combined (HIIT + high load resistance + inspiratory muscle training (IMT)) program over a period of 4.5 years.

## 2. Experimental Section (Case Report)

The patient is a post-menopausal woman aged 52 years at the start of the study (height 162 cm; weight, 54 kg; body mass index, 20.6 kg·m^−2^), diagnosed in 2007 with the most severe presentation of systemic sarcoidosis according to the American Thoracic Society criteria—stage IV—indicating irreversible scarring in the lungs (pulmonary fibrosis) [21], in the Pulmonary Unit of the Hospital Universitario de Gran Canaria Doctor Negrín (Canary Islands, Spain).

**Figure 1:**
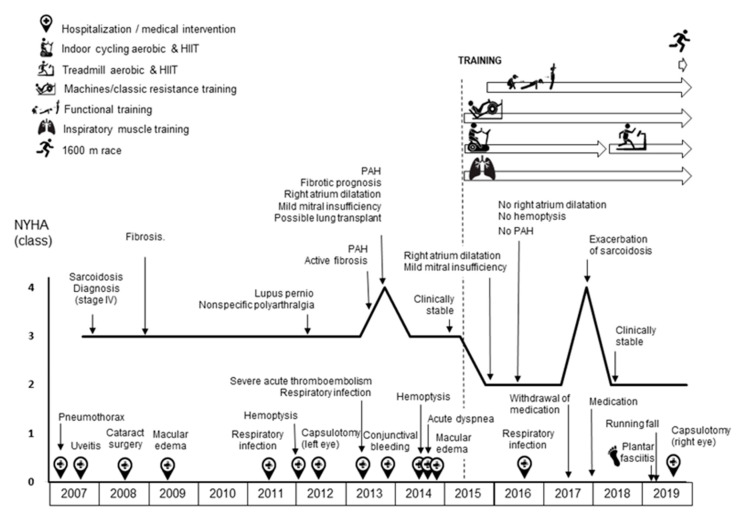
Graphical Abstract. Clinical and functional evolution of the patient since diagnosis of sarcoidosis. Abbreviations: HIIT, high-intensity interval training; NYHA, New York Heart Association; PAH, pulmonary arterial hypertension.

Beyond pulmonary involvement (which also included bilateral peribronchial and mediastinal calcified lymphadenopathy and iatrogenic pneumothorax), she had ocular, dermal, and articular symptoms (Figure 1). She has been treated with corticosteroids since diagnosis and has a history of recurrent exacerbations and hemoptysis episodes of infectious origin. In 2013, she had an episode of severe acute pulmonary thromboembolism and was diagnosed with moderate PAH, diastolic atrial dilation, and mild mitral insufficiency; she was classified as New York Heart Association (NYHA) class IV. For this reason, she was also treated with anticoagulants and was considered as a candidate for eventual lung transplantation. Two years later, despite being clinically stable and having improved her status such as to be classified as NYHA class III, the patient reported a marked limitation in physical activities. She also reported that she needed to rest every ~100 m when walking in order to recover from the associated dyspnea, with recurrent episodes of hemoptysis also triggered by exertion.

She practiced athletics from the age of 17 to 20 years and adopted an essentially sedentary lifestyle since age 23 until starting our intervention—except for two 12-week respiratory rehabilitation programs performed 4 and 3 years, respectively, before the start of our exercise training intervention.

### 2.1. Assessments

All assessments were performed every 6 months (June and December) at the Exercise Physiology Laboratory at Hospital 12 de Octubre (Madrid, Spain), which she first visited in June 2015. After we studied her medical history in depth (with one of us (A.L.) being a physician with solid expertise in the implementation of exercise interventions, including in-hospital programs, for patients with numerous disease conditions such as PAH [22]), she underwent a battery of physical capacity assessments (as described below) every six months. She gave her written consent to participate in the study and to have her data published. The study was conducted in accordance with the Declaration of Helsinki, and the protocol was approved by the Ethics Committee of the aforementioned institution (approval number: 13/377).

First, we measured maximum inspiratory pressure (MIP) (RPM, Micro Medical Inc., Chatham, Kent, UK). Due to the history of recurrent hemoptysis (some during spirometry) and the fact that performing forced expiration is known to increase pulmonary capillary pressure [23], we refrained from performing spirometry or maximum expiratory pressure assessments.

Thereafter, the patient performed a cycle ergometer (Ergometrics 900, Ergoline, Barcelona, Spain) exercise test until volitional exhaustion, during which blood pressure (BP), heart rate (HR), 12-lead ECG, peripheral oxygen saturation (SpO_2_) (TRUSAT 3500, General Electric Oy; Helsinki, Finland) and gas exchange data (CPX Ultima, Medical Graphics Corporation, St Paul, MN, USA) were continuously monitored. We followed a published protocol [24] with slight modifications. Thus, after a 2-min resting period on the ergometer, the patient pedaled for 2 min at a cadence of 60–65 rpm without resistance (0 watt). The workload was then increased by 1 watt every 6 s (averaging 10 watts/min) until exhaustion or until SpO_2_ decreased below 90%. Other criteria for test termination were chest pain, ECG signs of ischemia, severe dyspnea disproportionate to the effort, tachyarrhythmias, or a drop in BP. The protocol also included a recovery phase, during which the patient pedaled for one minute at 10 watts and one minute without resistance, before finally resting for one minute on the ergometer to prevent post-exercise desaturation. Gas-exchange parameters (10-s averages) where collected for the determination of the peak value of oxygen uptake (VO_2_peak) reached during the tests, as well as the ventilatory threshold (VT) and respiratory compensation threshold (RCP, also termed “second ventilatory threshold”). The VT corresponded to the workload at which the ventilatory equivalent for oxygen (ventilation (VE∙VO_2_^−1^) starts to increase with no concomitant increase in the ventilatory equivalent for carbon dioxide (VE·VCO_2_^−1^) and with departure from linearity of VE. The RCP was determined as the workload at which both VE·VO_2_^−1^ and VE·VCO_2_^−1^ increase together with a decrease in end-tidal pressure of carbon dioxide [25].

In the first visit only, the patient performed a circuit strength training session using basic machines (chest press, leg press, lateral pull down, and abdominals), during which we monitored SpO_2_ to analyze her SpO_2_ response (and eventual drops of this variable) during and after each set of exercises.

After the first year of training, we incorporated body composition analysis (dual X-ray absorptiometry; Hologic Discovery QDR Series, Software Physician’s Viewer, APEX System Software Version 3.1.2. Bedford, MA, USA) to the battery of assessments. Body mass composition was calculated from a full body study scan, and a specific analysis of bone density in the spine (L_1_-L_4_) and femoral neck was performed as described [26].

### 2.2. Exercise Training Intervention

The patient underwent a combined (HIIT + high load resistance + IMT) program for a total of 4.5 years in her local fitness center under the supervision of a fitness specialist. The patient generally trained five to six days/week. This included two sessions of strength training using machines, two sessions of strength training using “functional exercises”, and two HIIT sessions. When she performed two sessions per day, this consistently included a HIIT and a functional training session, respectively.

The HIIT program is detailed in Table 1. Heart rate and SpO_2_ (TRUSAT 3500, General Electric Oy; Helsinki, Finland), and rating of perceived exertion (RPE, on a 0 to 10 scale) were recorded in each session. During the first three years all the sessions were performed on a cycle-ergometer (S-UBx, Star Trac, USA). To prevent eventual abrupt decreases in SpO_2_ after exertion, each session ended with a cool-down phase (6 min of pedaling at 15 watts with a cadence of 60 rpm, followed by 2 min during which resistance was gradually reduced to 0). In the last phase (1.5 years) of the program, the HIIT sessions were performed on a treadmill (S-TRc, Star Trac, CA). The transition from HIIT cycle-ergometer to HIIT treadmill sessions served two purposes: (i) to apply a more functional exercise modality with a greater transference to daily life activities; and (ii) to increase the impact load associated with exercise in order to prevent—or at least attenuate—the loss of bone mineral density that is known to be associated with both post-menopause and corticosteroid treatment [27,28].

In the last month of the follow-up, and with the goal of further motivating the patient, she completed a one-mile running race at an average speed of 6.4 km/h (range 6–7) with SpO_2_ consistently >90%. Of note, this race speed was similar to the one corresponding to the active rest periods between intervals in the treadmill HIIT sessions (Table 1).

Strength training program is detailed in Table 2. It included the same circuit on machines (chest press, leg press, lateral pull down, and abdominal) as on the first visit and was performed twice weekly. The load was progressively increased to 4 series of 7 repetitions in the sixth month. Thereafter, chest press was replaced by a modified chest fly exercise, performed with the trunk titled ~10° forward and with grip over the shoulder (Video S1), and the lateral pull down was changed to a behind-the-neck lateral pull down executed with the trunk tilted 10° forward (Video S2). These variations were intended to expand the rib cage against the patient’s manifest dorsal hyperkyphosis.

Thereafter, eccentric Russian belt squat (Video S3) and, also, clean and jerk exercises were added to the gym machine routine. From the seventh month, and on different days, a functional exercise routine (step-full squats (Video S4), lunges (Video S5), step-squat jumps (Video S6), and burpees (Video S7)) was added and performed twice weekly (Table 2). HR and SpO_2_ were continuously monitored in all sessions, as was RPE, using the 0–10 OMNI Resistance Exercise Scale [29] at the end of each exercise. The resistance training scheme aimed to allow the patient to achieve the highest maximum training volume, combining high loads and low repetitions while keeping SpO_2_ above 90% (or at least allowing a rapid recovery of this variable to baseline levels in case it fell below 90%). This combination was selected since high loads increase maximum force more than low loads [30], and a low number of sets and repetitions have been shown as effective in increasing strength as a combination of a high number of sets and repetitions in post-menopausal women [31].

IMT was done using a mechanical pressure threshold device (Powerbreathe Plus Low Resistance, Powerbreathe Spain-Biocorp Europa; Andoain, Spain). It started with two sessions (morning and evening) of 10 maximum inspirations at 42% of MIP, five days a week, increasing gradually to 30 inspirations per session in the sixth month. Thereafter, the number of repetitions was maintained, and the inspiratory load was progressively increased depending on the patient’s intensity perception. She was asked to gradually increase the load of the inspiratory device so that it was always difficult, but not impossible, for her to do all the inspirations. After the second year, she performed two sessions of 60 inspirations per day, increasing the inspiratory load as described.

## 3. Results

During the study period the patient’s functional status improved from NYHA III (at the start of the intervention, in the middle of year 2015) to II (at the conclusion of the 4.5-year intervention, end of year 2019). At the end of 2016 and throughout 2017 several breaks in training occurred due to exacerbation of the disease likely associated with the progressive reduction in corticoid dosage (since clinical severity had decreased) until the withdrawal of the drug in May 2017 and reintroduction again at the start of 2018 (due to exacerbation of symptoms) (Figure 1). In addition, during January–March 2019, the patient developed plantar fasciitis that led to the suspension of HIIT during this period. Although in some months SpO_2_ dropped below 90% after the HIIT sprints, it consistently returned to baseline levels very quickly (within 10–15 s).

Strength training was affected by medication withdrawal in the same way as HIIT (Table 2). Plantar fasciitis forced the suspension of the dynamic exercises, and the replacement of burpees with static planks. However, machine strength training was maintained throughout the duration of injury. As for the HIIT sessions, in those cases where SpO_2_ dropped below 90% it normalized very quickly (10–15 s).

The inspiratory load increased from 20 to 40 inspirations per day at 42% MIP of restriction to 120 inspirations per day at 85% of MIP, causing a 19% increase in MIP (reaching 127 cm H_2_O). In addition to the interruptions of IMT due to the worsening of her condition after the medication withdrawal, IMT was suspended in March 2019 due to rib pain after a fall while running on the treadmill.

The effects of training in cardiopulmonary exercise testing data are shown in Table 3. Notably, VO_2peak_ and peak pulmonary ventilation increased by 44% and 60%, respectively, from the start to the end of the study period. Table 4 summarizes total and regional bone mineral density (BMD), lean and fat mass values during the study.

## 4. Discussion

The main finding of our study was that a combined (HIIT + strength + IMT) program, applied for 4.5 years in a patient with sarcoidosis (stage IV) and pulmonary involvement and treated with corticoids for 12 years, contributed—at least partly—to increase functional capacity (improving a class in the NYHA classification) as well as cardiorespiratory fitness (CRF, i.e., increase in VO_2_peak from barely ~6 metabolic equivalents (MET) at the start of the study to 8.3 MET at the end). This is an important finding because the VO_2_peak level reflects the synergistic action of pulmonary, cardiovascular and muscle tissue via a suite of physiological actions that effectively transport and deliver oxygen from the atmosphere to mitochondria in working muscles [32]. CRF, as determined by VO_2_peak, is a strong prognostic factor of morbimortality, particularly in relation to cardiometabolic diseases [33]. Moreover, it is of clinical importance to surpass the eight-MET threshold, and in fact adults with a VO_2_peak above this level have a reduced cardiovascular risk [34]. Of note, the increase in CRF was corroborated—being in fact of the same relative magnitude—when VO_2_peak was expressed in either absolute units (mL·min^−1^) or relative to body mass (mL·kg^−1^·min^−1^). Furthermore, the intervention was safe, with no persistent SpO_2_ drops or syncope episodes.

A reduced maximum exercise capacity is related to frailty, conceptually defined as a physical vulnerability to stressors. Frailty is considered an important phenotype in lung transplant candidates independent of the severity of the disease [35]. On the other hand, 10% of patients with sarcoidosis progress to fibrotic lung disease and may be in need of lung transplant [18]. In this regard, although lung transplantation was originally proposed as a future option for our patient, the remission of right atrium dilatation, mild mitral insufficiency, and PAH, together with improvements in NYHA class and CRF made it possible to discard transplant by the end of first year of the intervention.

HIIT induces both peripheral (increased muscle capillary density and mitochondrial content) and central (increased cardiac output) cardiovascular adaptations [36]. Furthermore, HIIT can increase alveolar capillary density through nitric oxide-induced vascular endothelial growth factor (VEGF) [37]. In fact, the HIIT protocol used here was designed to allow the accumulation of as many training loads near VO_2_peak as possible without drops in SpO_2_. One the one hand, and likely related to this, we observed an improvement in ventilatory efficiency, as expressed in VE·VO_2_^−1^ at submaximal intensities (which decreased by 32% and 14% at VT and RCP, respectively, with the latter value similar to that of healthy subjects) [38]. Improved ventilatory efficiency might have contributed, at least in part, to the observed increase in the patient’s functional independence and the absence of high SpO_2_ drops in her daily life activities.

IMT has been shown to improve exercise capacity, functionality, respiratory muscle strength, dyspnea, and fatigue in patients with sarcoidosis [39]. These improvements can be explained by the resulting increase in respiratory muscle strength [40] and are reflected by increases in MIP and peak ventilation during exercise. IMT-induced improvements in lung compliance can increase the surface of gas exchange [41]. Furthermore, IMT decreases the oxygen cost of breathing [41], thereby reducing the fatigue-induced respiratory metabolic reflex with subsequent increases in blood flow to locomotor muscles [42].

Resistance training may also have played a role in increasing the VO_2_peak (mL/min) and improving the patient’s NYHA functional class. The increase in maximum strength may have allowed her, at least partly, to maintain a higher training work rate and increase the ability to cope with—and adapt to—cumulative HIIT sessions. Resistance training is known to improve work economy [43] and thus decreases the percentage of work demanded by daily life activities (e.g., walking, climbing stairs, and carrying bags); it thus allows performing these submaximal actions with less oxygen cost [44]. Muscle mass could have been affected by the patient’s menopause and prednisone treatment. Menopause frequently causes sarcopenia, which is a syndrome characterized by a progressive and generalized loss of skeletal muscle mass and strength [45]. Likewise, corticoid treatment reduces protein synthesis and increases muscle protein catabolism [46], especially in type II fibers [47]. Although muscle mass contributes to improvements in strength, our approach was actually designed to improve muscle strength through neural factors, by increasing motor unit recruitment and firing frequency of active motor units without the need for major increases in muscle mass [48]. This is in fact why high loads were used, as they increase neural adaptations compared with lower loads [49]. In this regard, a previous study applied high-load strength training to patients with sarcoidosis for 12 weeks [13]. Although the relatively short training duration of that study suggests that the strength gains noted were likely due to neural factors, the absence of muscle mass measurements and the persistence of adaptations five months after the training period makes it difficult to quantify the neural factor influence. Our results are, however, in line with the Kullberg et al. study regarding the feasibility, safety and effectiveness of high-load resistance training in patients with sarcoidosis.

It is widely known that bone and lean mass decrease with age, particularly in menopausal women due to hormonal changes [50]. Corticoids also accelerate the loss of bone mass [51] whereas the opposite effect is seen with exercise, which is in fact recommended for the prevention of osteoporosis and osteopenia [52]. Our results would support the beneficial effect of exercise on bone health, since we observed only a slight decrease in BMD over the three-year period despite the post-menopausal status of the patient and the corticoid treatment she received for years. Indeed, analysis at the end of the follow-up revealed only mild osteopenia of the femoral neck and normal bone health at the spine (T-score = −1.1 and −0.8, respectively), indicating that our patient was above the average for women of her age (Z-score range = 0.1–0.4). Similarly, the exercise intervention probably had a certain beneficial effect on lean mass, which decreased only in the last measurement likely due to the reduction in the volume of strength training, which dropped from 4 to 2 days/week after a plantar fasciitis.

The NYHA class improvement, together with the well-known deleterious effects of chronic corticoid treatment led to the progressive withdrawal of this drug after 1.5 years from the start of the training program. With the exception of the sarcoidosis flare-up caused by medication withdrawal, only one sarcoidosis-related hospital admission (due to respiratory infection) and one medical intervention (capsulotomy) related to sarcoidosis occurred in the 4.5 years. Adverse events related to training were a running fall from the treadmill (not associated with arterial oxygen desaturation) and plantar fasciitis. Despite these episodes, the patient maintained her adherence to the training program due to the positive impact that the improvement in QoL and wellbeing early during the intervention (in the first few months) had on her level of motivation, as well as to her strong self-discipline.

The present study has several limitations that should be considered. First, as this is a case report, our preliminary results in only one patient must be interpreted with caution and cannot be generalized to all patients with sarcoidosis. In this regard, it would be interesting to conduct a randomized controlled trial with a long-term intervention like the present one, although this type of study might be very difficult to conduct. Second, the 1RM was not evaluated, and thus although an increase in load (kg) was observed with resistance training, we could not quantify the magnitude of the actual improvement in maximum strength. Finally, diet was not controlled.

## 5. Conclusions

In summary, the results of this study suggest that a long-term combined (HIIT + strength + IMT) exercise intervention might have contributed, at least partly, to safely improve CRF and functional capacity (NYHA class) in a patient with severe thoracic sarcoidosis. Our results also suggest that the intervention could have acted as a coadjuvant treatment that helped to partially reduce the severity of associated pathologies (cardiac involvement and PAH) and attenuate the loss of BMD and muscle mass caused by menopause and chronic corticoid treatment. Randomized controlled trials are needed to confirm our preliminary findings.

## Figures and Tables

**Table 1:** High-intensity interval training program.

Period	Days/Week	Exercise Mode	Total Session Duration (Min)	Between Intervals				Intervals/Sprints				
				Average Internal Load	Average HR (Beats·Min^−1^)	Average %HRmax	Average SpO_2_ (%)	Number × Duration/Recovery	RPE (0–10)	Average HR (Beats·Min^−1^)	HR (%HRmax)	Average SPO_2_MIN (%)
**July–September 2015**	4	Cycling	30	50% (VT-RCP)	125	74	96	10–15 × 8 s/2–3 min	9	130	77	93
**October–December 2015**	3–4	Cycling	40–60	50%(VT-RCP)	132	79	94	14–30 × 8 s/2–3 min	8	135	80	93
**January–March 2016**	3–4	Cycling	60	50% (VT-RCP)	135	81	95	30 × 5–10 s/1 min	7	141	84	91
**April–June 2016**	3	Cycling	60	50% (VT-RCP)	125	75	94	30 × 5 s/1 min	7	135	81	91
**July–September 2016**	3	Cycling	60–45	50% (VT-RCP)	125	75	95	30–23 × 10 s/2 min	7	135	81	90
**October–November 2016**	3–4	Cycling	45	50% (VT-RCP)	125	75	94	23 × 10 s/2 min	7	135	81	90
**December 2016**	Decrease in medication (corticosteroids)											
**January–March 2017**	3	Cycling	45–60	50% (VT-RCP)	135		94	23–30 × 10 s/2 min	8	145	87	90
**April 2017**	2	Cycling	60	50% (VT-RCP)	135	81	94	30 × 10 s/2 min	8	145	87	89
**May–June 2017**	Progressive withdrawal of medication											
**July–September 2017**	2–3	Cycling	30–60	50% (VT-RCP)	130	78	94	15–30 × 10 s/2 min	7	145	87	90
**October–November 2017**	3	Cycling	45–60	50% (VT-RCP)	135	81	94	23–30 × 10 s/2 min	7	145	87	90
**December 2017**	Exacerbation of sarcoidosis											
**January–March 2018**	2	Cycling	60	50% (VT-RCP)	135	82	94	30 × 10 s/2 min	6	143	87	89
**April–June 2018**	3	Running	30	7 km·h^−1^	135	82	90	12 × 15 s (15 km·h^−1^)/2.5 min	9	140	85	89
**July–September 2018**	2–3	Running	40–60	7 km·h^−1^	140	85	92	16–24 × 15 s (15 km·h^−1^)/2.5 min	9	155	94	91
**October–December 2018**	2	Running	40–60	7 km·h^−1^	142	86	93	16–24 × 15 s (15–18 km·h^−1^)/2.5 min	9	165	100	89
**January–March 2019**	Plantar fasciitis											
**April–June 2019**	2	Cycling	30–45	50% (VT-RCP)	135	82	93	10–23 × 8 s/2–3 min	5	145	88	89
**July–September 2019**	2	Running	45	7 km·h^−1^	135	82	94	23 × 10 s (15 km·h^−1^)/2 min *	9	155	95	85
**October–December** **2019**	2	Running	50–45	7 km·h^−1^	135	82	94	25–23 × 10 s (15 km·h^−1^)/2 min **	9	155	95	85

Abbreviations: HR, heart rate; HRmax, age-predicted maximum heart rate (220 minus age, in years); HRpeak, peak heart rate achieved after sprint; RPE, rating of perceived exertion in Borg scale (0–10); SpO_2_, peripheral oxygen saturation; SpO_2_min, minimum value of SpO_2_ after a sprint; _50%(VT-RCP)_, internal (i.e., heart rate-based) work load equidistant between the ventilatory threshold and the respiratory compensation point. Symbols: * 3kg weighted vest; ** 4 kg weighted vest.

**Table 2:** Resistance training program.

Period	Days/Week	Classic Resistance Training						Days/Week	Functional Training			
		Modified Chest Fly Machine *	Leg Press	Modified Behind the Neck Lat Pulldown **	Planks ***	Russian Belt Squat	Clean & Jerk/Split		Step-Full Squat	Lunges	Squat Jumps	Burpees
**July–September 2015**	2	3 × 5–10 kg (8–92%)	3 × 6–10/30 kg (6–94%)	3 × 5–15 kg (7–88%)	2/4 × 6 (8–94%)							
**October–December 2015**	2	4 × 7–10 kg (8–91%)	4 × 7–30/40 kg (6–94%)	4 × 7–15 kg (7–88%)	4 × 7 (7–92%)							
**January–March 2016**	2	4 × 8−15 kg (9–91%)	4 × 8−50 kg (6–95%)	4 × 8–25 kg (8–88%)	4 × 8 (7–94%)			2	3 × 15 (8–90%)	3 × 15 (5–93%)	3 × 1/12 (7–90%)	
**April–June 2016**	2	4 × 8−15 kg (9–92%)	4 × 8−50 kg (6–95%)	4 × 8–25 kg (8–88%)	4 × 8 (7–94%)	4 × 8–10 kg (9–90%)		2	3 × 15 (6–90%)	3 × 15 (5–93%)	3 × 12 (8–90%)	3 × 8 (9–89%)
**July–September 2016**	2	4 × 8−15 kg (8–92%)	4 × 8 −50/60 kg (7–95%)	4 × 8–25 kg (7–88%)	4 × 8 (7–94%)	4 × 8–10 kg (8–90%)		2	3 × 15 (5–90%)	3 × 15 (5–94%)	3 × 12 (7–88%)	3 × 8 (9–88%)
**October–December 2016**	Decrease in medication (corticosteroids)											
**January–March 2017**	2	4 × 8−15 kg (8–92%)	4 × 8 −50/60 kg (8–95%)	4 × 8–25 kg (8–85%)	4 × 8 (7–94%)			2	0/3 × 15 (6–90%)	0/15 (5–92%)	0/3 × 12 (8–88%)	0/3 × 0/8 (9–85%)
**April 2017**	2	4 × 8−20 kg (9–90%)	4 × 8 −60 kg (8–94%)	4 × 8–25 kg (8–85%)	4 × 8 (7–94%)	4 × 8–10 kg (8–88%)		2	3 × 15 (6–90%)	15 (5–92%)	3 × 12 (8–88%)	
**May–June 2017**	Progressive withdrawal of medication											
**July–September 2017**	0–2	4 × 8−20 kg (8–90%)	4 × 8 −60 kg (7–94%)	4 × 8–25 kg (8–85%)	4 × 8 (7–95%)	4 × 8–10 kg (8–88%)		0–2	0/3 × 12 (8–88%)	0/3 × 12 (9–88%)	0/3 × 10 (9–88%)	
**October–December 2017**	Exacerbation of sarcoidosis											
**January–March 2018**	2	4 × 8−20/15 kg (7–90%)	4 × 8 −60/70 kg (7–94%)	4 × 8–20 kg (7–89%)	4 × 8 (7–95%)	4 × 8 –10 kg (8–90%)	4 × 6–7.5 kg (9–88%)	2	3 × 15 (6–94%)	3 × 15 (5–94%)	3 × 12 (6–88%)	
**April–June 2018**	2	4 × 8−15 kg (8–88%)	4 × 8−60 kg (7–94%)	4 × 8–20 kg (7–89%)	4 × 8 (7–95%)	4 × 8–8 kg (8–89%)	4 × 6–7.5 kg (9–88%)	2	3 × 15 (5–94%)	2 × 15 (5–94%)	2 × 12 (5–90%)	
**July–September 2018**	0–2	2/4 × 8−20 kg (7–92%)	4 × 8−70 kg (7–92%)	2/4 × 8–25 kg (8–88%)	2/4 × 8 (7–95%)	2/4 × 8–8 kg (7–92%)	4 v 6–7.5 kg (9–88%)	1–2	3 × 15 (5–94%)	0/2 × 15 (5–94%)	0/2 × 12 (5–90%)	
**October–December 2018**	0–2	2 × 8−20 kg (7–92%)	4 × 8−70 kg (7–94%)	2 × 8–20 kg (7–88%)	2 × 8 (7–95%)	2 × 8–8 kg (7–92%)	4 × 6–7.5 kg (9–86%)	1–2	3 × 15 (5–94%)	0/3 × 15 (5–95%)	0/3 × 12 (5–90%)	0/3 × 10 (9–85%)
**January–March 2019**	1–2	2/3 × 8−20 kg (9–88%)	2/3 × 8−70 kg (9–93%)	2/3 × 8–25 kg (9–88%)	2/3 × 8 (8–95%)	2/3 × 8–8 kg (8–90%)	Plantar fasciitis					
**April–June 2019**	1	2/3 × 8−20 kg (9–88%)	2/3 × 8−70 kg (8–93%)	2/4 × 8–25 kg (9–85%)	2/4 × 8 (8–93%)	2/3 × 8–8 kg (9–90%)		1	3 × 15 (8–90%)	3 × 15 (8–92%)	3 × 12 (7–90%)	4 static planks
**July–September 2019**	1	4 × 8−20 kg (8–92%)	4 × 8−70 kg (7–94%)	4 × 8–25 kg (7–85%)	4 × 8 (9–88%)			1	3 × 15 (8–90%)	3 × 15 (8–90%)	3 × 12 (6–90%)	4 static planks
**October–December 2019**	1	4 × 8−20 kg (7–92%)	4 × 8−70 kg (7–95%)	4 × 8–25 kg (7–85%)	4 × 8 (9–88%)			1	3 × 15 (9–90%)	3 × 15 (8–90%)	3 × 12 (6–90%)	4 static planks

Classic and functional exercises were done twice a week in different days. Data are expressed in sets x reps-kg (RPE–SpO_2_%). Data are expressed sets/sets, reps/reps or kg/kg in progressive increases during a 3-months period. Burpee: dynamic combination of stand up, squat down, plank, jump out and push up. Burpees were replaced by 30-s static planks after plantar fasciitis. RPE: rating of perceived exertion in OMNI (0–10) scale in static/machine exercises and in Borg (0–10) scale in dynamic exercises. SpO_2_: Lowest oxygen saturation (%) after a set. * chest press in 2015, then changed to chest fly (trunk tilted ~10° forward and with grip over the shoulder) 2016–2019; ** trunk tilted 5° forward, with wide grip; *** abdominal floor exercises (crunch, oblique crunch, crunch clap, scissors) in 2015, then changed to plank exercises (plank, plank to push up, side plank, and raised-leg plank) 2016–2019.

**Table 3:** Results of cardiopulmonary exercise testing.

Variables	Pretraining	December 2015	June 2016	December 2016	June 2017	December 2017	June 2018	December 2018	June 2019	December 2019	Change from Baseline to 4.5 Years Later (%)
Peak values											
VO_2_peak (mL·kg^−1^·min^−1^)	20.1	22.4	30.2	23.6	24.4	25.2	28.9	28.3	26.6	28.9	+44%
VO_2_peak (mL·min^−1^)	1086	1212	1631	1275	1316	1360	1560	1550	1502	1561	+44%
PPO (watts)	100	119	123	130	125	120	130	127	130	124	+24%
HRpeak (bpm)	166	164	164	167	162	170	170	166	170	172	+4%
HR (% HRmax)	99	98	98	100	98	102	103	100	104	105	+6%
VEpeak (L/min)	62	59	73	65	83	87	81	97	77	99	+60%
SpO_2_peak (%)	93	91	91	88	89	88	89	89	89	91	−2%
VT											
VO_2_ (mL·kg^−1^·min^−1^)	11.7	14.2	18.7	11.2	12.8	15.4	17.9	11.7	15.6	15.1	+29%
PO (watts)	47	57	66	35	47	50	47	41	60	42	−11%
VE·VO_2_^−1^	38	29	31	28	31	27	29	25	28	26	−32%
VE·VCO_2_^−1^	39	39	35	35	39	33	38	35	34	38	−3%
RCP											
VO_2_ (mL·kg^−1^·min^−1^)	17.4	18.1	26.6	19.8	19.3	21.2	24.1	22.3	18.3	23.9	+37%
PO (watts)	77	87	103	106	89	87	83	102	95	90	+17%
VE·VO_2_^−1^	42	42	35	36	36	35	35	36	32	36	−14%
VE·VCO_2_^−1^	36	37	33	32	35	32	36	32	33	37	+3%

Abbreviations: HR, heart rate; HRmax (age-predicted maximum heart rate, i.e., 220 minus age, in years); HRpeak, peak heart rate; PO, power output; PPO, peak power output; RCP, respiratory compensation point; SpO_2_, peripheral capillary oxygen saturation; VE·VO_2_^−1^, ventilatory equivalent for oxygen. VE·VCO_2_^−1^, ventilatory equivalent for carbon dioxide. VEpeak, peak ventilation; VO_2:_ oxygen uptake; VO_2_peak, peak oxygen uptake; VT, ventilatory threshold.

**Table 4:** Results of body composition.

Variables	Oct.	June	December	June	December	June	December	June	December	Change from Baseline to 3.5 Years Later (%)
	2007	2016	2016	2017	2017	2018	2018	2019	2019	
BMD (g·cm^−2^)										
Whole body		1.11	1.07	1.07	1.05	1.06	1.04	1.02	1.06	−4.5
Subtotal body		0.91	0.89	0.89	0.87	0.88	0.86	0.86	0.89	−2.2
Pelvic		1.09	1.06	1.06	1.06	1.07	1.01	1.05	1.10	0.9
Arms (mean)		0.67	0.65	0.65	0.64	0.65	0.65	0.64	0.65	−3.0
Legs (mean)		1.10	1.09	1.08	1.06	1.04	1.06	1.06	1.06	−3.6
Lumbar (mean L_1_–L_4_)	1.24	0.98	0.97	0.96	0.94	0.92	0.92	0.93	0.96	−2.0
T-Score spine	0.4	−0.6	−0.7	−0.8	−0.9	−1.2	−1.1	−1.1	−0.8	
Z-Score spine	1.1	0.4	0.3	0.3	0.1	−0.1	0.0	0.1	0.4	
Femoral neck	1.01	0.83	0.76	0.81	0.84	0.76	0.73	0.74	0.73	−12.0
T-Score femoral neck	0.2	−0.1	−0.8	−0.3	−0.1	−0.8	−1.2	−1.0	−1.1	
Z-Score femoral neck	0.7	0.8	0.2	0.7	1.0	0.2	0.1	0.1	0.1	
Lean mass (kg)										
Whole body		40.4	38.5	39.6	38.5	40.4	39.2	38.4	35.8	−11.4
Subtotal body		35.6	33.6	34.6	33.7	35.5	34.4	35.4	33.0	−7.3
Trunk		19.0	17.7	18.6	17.5	18.6	18.9	19.4	17.2	−9.5
Arms (mean)		2.0	1.9	2.0	1.9	2.1	1.9	1.8	1.9	−5.0
Legs (mean)		6.3	6.1	6.1	6.2	6.4	5.9	6.3	6.0	−4.8
Fat mass (kg)										
Whole body		10.3	12.9	12.9	12.1	13.9	14.1	16.3	15.9	54.4
Subtotal body		9.6	12.2	12.1	11.4	13.2	13.3	15.6	15.2	58.3
Trunk		3.7	4.9	4.7	4.6	5.3	5.4	6.2	6.3	70.3
Arms (mean)		0.3	0.4	0.4	0.4	0.5	0.5	0.7	0.6	100
Legs (mean)		2.6	3.2	3.3	3.0	3.5	3.4	4.0	3.9	50
Fat mass (%)		20.3	26.0	24.6	23.9	25.6	26.4	28.8	29.7	46.3

Abbreviations: BMD, bone mineral density.

## Supplementary Materials

The following are available online at https://www.mdpi.com/1660-4601/17/24/9512/s1, Video S1: Modified Chest Fly exercise, Video S2: Modified Lat Pull Down exercise, Video S3: Russian Belt Squat exercise, Video S4: Step-full squat exercise, Video S5: Lunge exercise, Video S6: Step-squat jump exercise, Video S7: Burpee exercise.
